# A subcellular sampling instrument allows spatial resolution of amyloid deposit-derived organelle-specific effects in microglia

**DOI:** 10.1038/s42003-024-07405-w

**Published:** 2025-01-03

**Authors:** Robert Subirana Slotos, Tinh Thi Nguyen, Ledjona Fiska, Kristina Friedland, Kristina Endres

**Affiliations:** 1 Department of Psychiatry and Psychotherapy, University Medical Center of the Johannes Gutenberg-University Mainz, Mainz, Germany; 2 Institute of Molecular Biology, Mainz, Germany; 3 Institute of Pharmacy, Johannes Gutenberg-University Mainz, Mainz, Germany; 4 Faculty of Computer Sciences and Microsystems Technology, Kaiserslautern University of Applied Sciences, Zweibrücken, Germany

**Keywords:** Diseases of the nervous system, Neurological disorders

## Abstract

Methodological developments in biomedical research are currently moving towards single-cell approaches. This allows for a much better spatial and functional characterization of, for example, the deterioration of cells within a tissue in response to noxae. However, subcellular resolution is also essential to elucidate whether observed impairments are driven by an explicit organelle. Here, we use the Single Cellome™ System SS2000 (Yokogawa) to investigate the local effects of Aβ plaque-like deposits (characteristic for Alzheimer’s disease) on mitochondria in the mouse microglial cell line SIM-A9. First, the specificity of subcellular extraction is demonstrated by detecting subcellular staining and RT-qPCR concerning marker genes by comparing nuclear and mitochondrial samples. Oxygen consumption and gene expression is then assessed in cells near and far from peptide deposits. Mostly, all analyses confirm the high specificity and integrity of the sampled material. In addition, impact of the peptide deposits occur concerning spatial distribution of the cells: e.g., oxygen consumption is only reduced in cells close to Aβ deposits but not in proximity to deposits of biologically inactive Aβ (scrambled) or in far distance. Moreover, a distance-related gene expression pattern occurs, demonstrating the local initiation of mitochondrial changes of microglia when approaching toxic peptide deposits.

## Abstract (web summary)

Aspiration of cellular content accompanied by confocal microscope-usage within the SS2000 allows investigation of organelle-enriched fractions and sheds light on selective effects of Aβ peptides.

## Introduction

Alzheimer’s disease (AD) can be divided into at least two entities—the sporadic and the familial. While it is quite clear that the genetic variant results from mutations or duplications in a limited number of genes (the amyloid precursor protein gene and the gamma-secretase complex genes PS1 and 2^1^), the underlying mechanisms of the sporadic variant are still not fully understood. However, there is a consensus in the field that a failure or overshooting of immune reactivity is an important contributor to pathogenesis^2^. Brain immune cells respond to oligomeric and deposited Aβ peptides, hallmarks of the disease, in a time-dependent manner: early contact induces phagocytic activity and activation^3,4^, while prolonged exposure may lead to senescence or overshooting activity that further damages the surrounding cells^5–7^. Aβ can bind directly to several receptors on the microglial surface. Triggering receptor expressed on myeloid cells 2 (TREM2), toll-like receptors (TLRs), and formyl peptide receptors (FPRs) can recognize extracellular Aβ as a ligand and other microglial proteins such as CD36 further contribute to the Aβ-evoked cellular response cascades (summarized in ref. ^8^). However, in addition to exposure time, there is also a spatial component, as few reports have described that cells close to deleterious peptide deposits respond differently from cells further away. For example, Olmedillas and colleagues^9^ used three virally transduced fluorophores to show that in the APPPS1 AD mouse model, the vicinity of plaques ( < 50 µm) contained a higher degree of migration-, death- and division-events of microglia as compared to behavior of all microglia in the AD brain or in the brains of wild type mice. They therefore identified the vicinity of plaques as hot spots of microglial turnover. By dividing the area around plaques into five concentric rings and performing spatial transcriptomics in AppNL-G-F AD model mice, it was shown that Apoe and CtsI are expressed exclusively in microglia within the inner ring (within 10 µm), which the authors defined as the amyloid plaque niche^10^. Other methodological approaches, such as laser capture microdissection, used for spatial studies, cannot achieve single cell resolution in most cases^11^. In addition, the use of a high-energy laser beam can damage the region of interest and introduce artefacts into the analytical processes. This may be even more relevant when attempting to sample subcellular structures with dimensions of only a few µm. Alternatively, organelles can be harvested by differential centrifugation or immunocapture after cell or tissue lysis^12,13^. These methods are time-consuming, which can lead to degradation of organelle function, and do not allow selection of samples from living cells representing a particular state (e.g. activated phenotype).

Single cell nanobiopsy methods have been established to collect small amounts of cellular material from individual cells, for example, based on scanning ion conductance microscopy^14^. The used syringes or nanopipettes comprise a diameter in the range of 150 to a few hundred nanometers and by this, prevent disruption of the cells (e.g^15^.). With such methods, pico- or femtoliters can be aspirated and longitudinal measures are feasible. Nevertheless, these custom-built devices need a high degree of technical expertize and sometimes lack supplies that can sustain cellular viability (e.g. extraction procedure performed at room temperature^15^). Moreover, some functional assays may require larger sampling volumes.

**Fig. 1:**
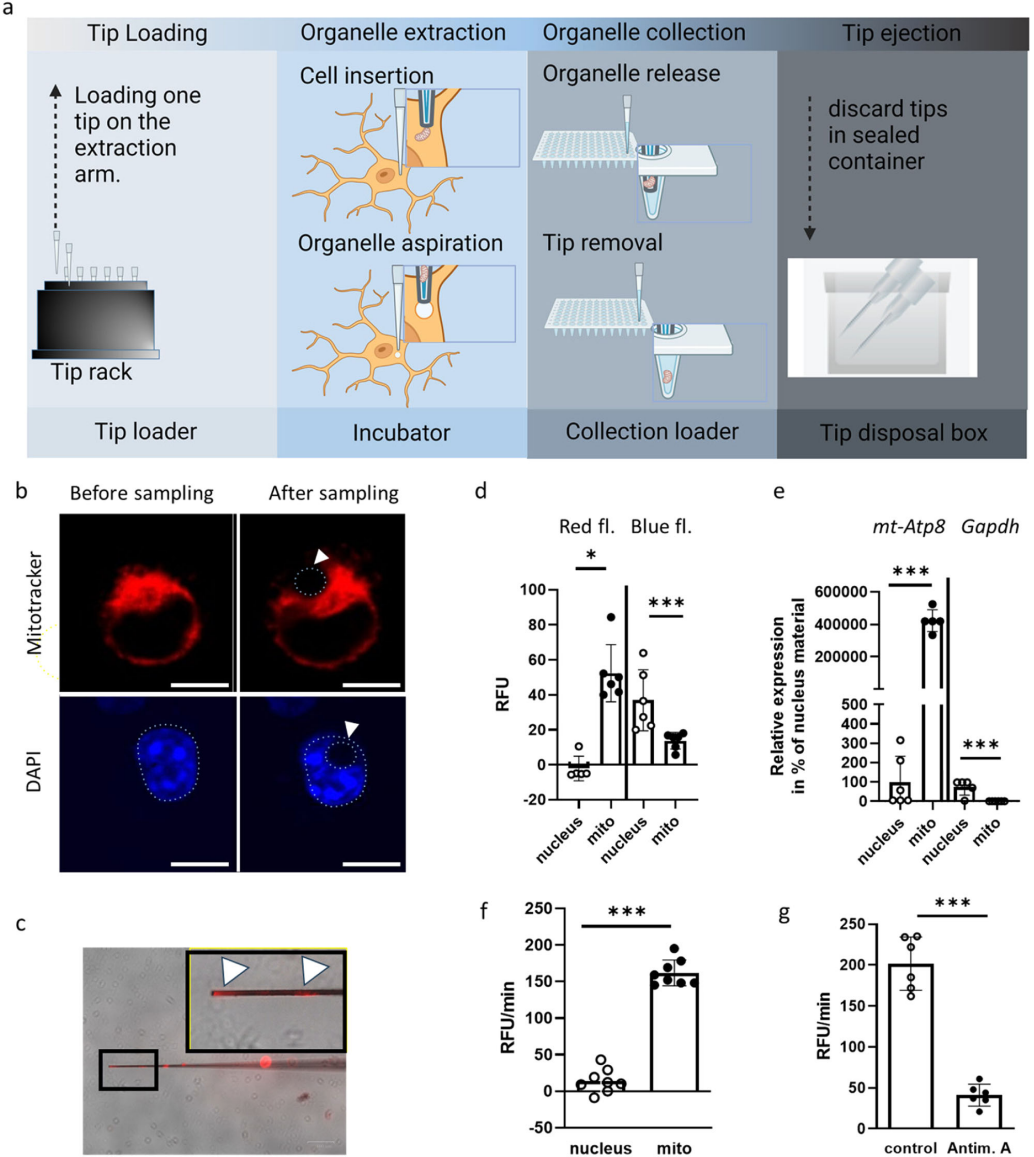
Subcellular compartment sampling of SIM-A9 cells. Cells were seeded at a density of 20,000 cells per well and incubated for 24 h. After staining with Lumitracker Mito Red and DAPI, cells were used for sampling of mitochondrial or nucleic material via tips with a diameter of 3 µm using the Single Cellome™ System SS2000. **a** A schematic of the instrument is shown. **b** Example images of cells before and after sampling are shown. The position of the nucleus is indicated by a dashed line, as is the area of aspirated material after sampling, which is also indicated by an arrowhead. Scale bar: 10 µm. **c** An example image of a tip with aspirated Lumitracker Mito Red-stained mitochondrial material is shown. A magnification of the part of the tip with extracted material (white arrowheads) is shown in the black box. **d** Both mitochondrial and nuclear material was extracted. Material from three cells was pooled to obtain one sample and fluorescence measured using 540 nm Exc/ 580 nm Em (red fl.) or 485 nm Exc/ 520 nm Em (blue fl.). Means of corrected relative fluorescence intensity (RFU) adjusted for background measurements (PBS) and SD are shown. **e** After fluorescence measurement, the subcellular compartment material was lysed and subjected to one-step RT-qPCR using primer pairs for Atp8 (mitochondrial gene product) or Gapdh (nucleic gene product). The values obtained for the mean of the nucleic samples were set to 100%. **f** Oxygen consumption was assessed immediately after sampling, with sample buffer as a negative control. Consumption was calculated as slope per minute (RFU/min) after correction for the slope of the negative control. Two independent experiments (*n* = 4 each) were performed. **g** Samples from the mitochondrial compartment with or without complex III inhibitor (antimycin A) were analyzed for oxygen consumption rate. Two independent experiments (*n* = 3 each) were performed. Unpaired two-tailed t-tests were used to compare the measurements obtained with Welch correction if needed (***p < 0.001; **p < 0.01; *p < 0.05).

To overcome these limitations, we tested the Single Cellome™ System SS2000 (Yokogawa), which promises to collect subcellular material by robotic aspiration through glass capillaries combined with confocal microscopy (Fig. 1a) allowing the visualization of amyloid deposits and mitochondria or nuclei in each cell before collecting organelles. We were able to demonstrate the applicability of the system by obtaining samples with the respective characteristics such as Lumitracker Mito Red-related fluorescence only in mitochondrial samples or specific detection of subcellular mRNA markers. Moreover, mitochondrial samples gained from close vicinity of Aβ deposits showed reduced oxygen consumption and a differential expression pattern compared to those collected in greater distance.

## Results

### Accuracy of organelle sampling

SIM-A9 cells were plated in a 96-well format and mitochondrial and nuclear material was collected using organelle-specific stains (Lumitracker Mito Red and DAPI). To assess the accuracy of mitochondrial and nuclear sampling, we visually followed the extraction process (Fig. 1b, c) and measured organelle-specific dye fluorescence as well as marker gene expression in the collected material (Fig. 1d, e). For example, mitochondrial material showed a significantly lower blue fluorescence signal compared to nucleic material (*p* = 0.0106), indicating the comparatively low but still DAPI-stainable DNA content in these organelles^16^. Expression of the nucleic marker gene Glyceraldehyde-3-phosphate dehydrogenase (*Gapdh*) was found exclusively in the nucleus with no measurable signals in mitochondrial material (*p* = 0.0008), whereas the mitochondrially encoded ATP synthase membrane subunit 8 (*mt-Atp8*) was almost exclusively expressed in mitochondria-derived samples (*p* = 0.0002). To assess the functionality of the isolated mitochondrial material, we analyzed oxygen consumption and obtained a tenfold higher consumption rate in mitochondrial material compared to nucleic material (Fig. 1f, *p* < 0.0001). This oxygen consumption was significantly inhibited by the administration of a complex III inhibitor (Fig. 1g, *p* < 0.0001).

### Assessment of gene expression pattern in mitochondria and nuclei in dependency from distance to peptide deposits

**Fig. 2:**
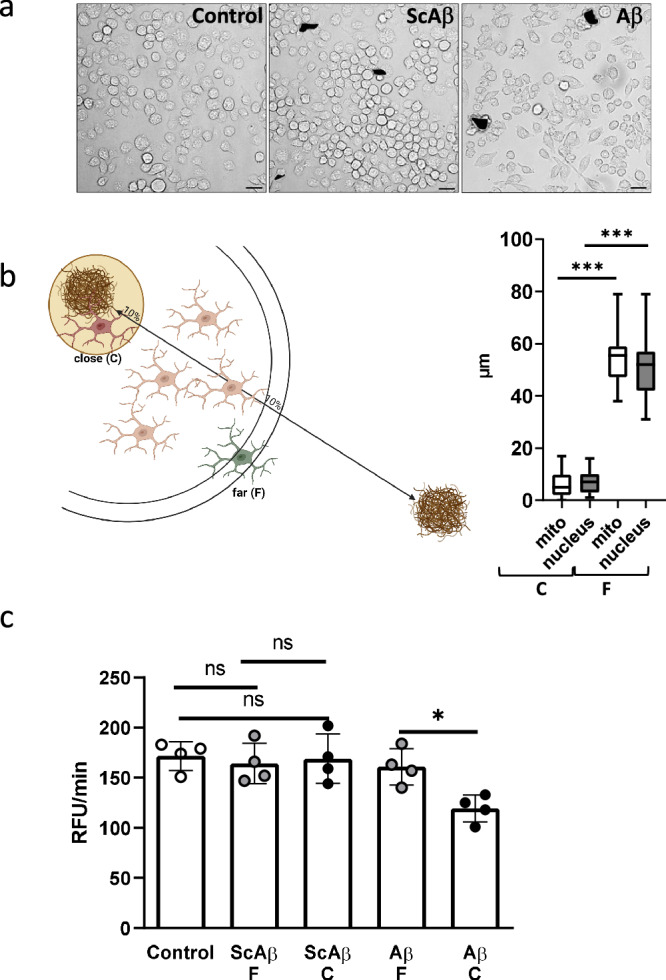
Spatial collection of organelle material and analysis of mitochondrial respiration from Aβ-deposit-exposed SIM-A9 cells. **a** Cells were exposed to pre-aggregated peptides or solvent control (PBS). Sample images were taken after 24 h. Scale bar: 25 µm. Peptide deposits have been artificially stained black for better visualization (original images provided in Suppl. Figure 1). **b** After 24 h exposure, mitochondrial and nucleic material was collected from cells in close proximity (close, C) to peptide deposits or further away (far, F) after staining of mitochondria as indicated in the scheme. Far and near distances were defined using the half-maximum distance between two neighboring peptide deposits, selecting the 10% distance near the deposit and the 90-100% of the radius away from the deposit. Distances to the edge of the deposits are expressed as mean + SD (*n* = 36 per group, values derived from all samples aspirated). Brown–Forsythe ANOVA test with Dunnett’s T3 multiple comparisons test was performed for statistical analysis (***p < 0.001). The box plot indicates min and max. **c** Oxygen consumption rate was measured in freshly sampled mitochondrial material (ScAβ: scrambled peptide; Aβ: Aβ peptide). Four independent experiments were performed (*n* = 4). Each sample consisted of material from three cells ejected in 6 µL buffer. Random samples were taken for the PBS control. Statistical analysis was performed by one-way ANOVA followed by Sidak’s multiple comparison post-test (*p < 0.05; ns, *p* > 0.05).

Having confirmed selective organelle sampling, we exposed SIM-A9 cells to Aβ-deposits to explore the applicability of subcellular sampling in a pathological environment. Cells were categorized according to their distance from a deposit (Fig. 2a, b). A scrambled peptide (Sc, lacking biological activity) was used as a control in addition to mere solvent administration control. Measurement of oxygen consumption in extracted mitochondria showed that only mitochondria from cells close to toxic Aβ-deposits and not to scrambled peptide aggregates showed a reduced respiration rate (Fig. 2c, *p* = 0.0126).

**Fig. 3:**
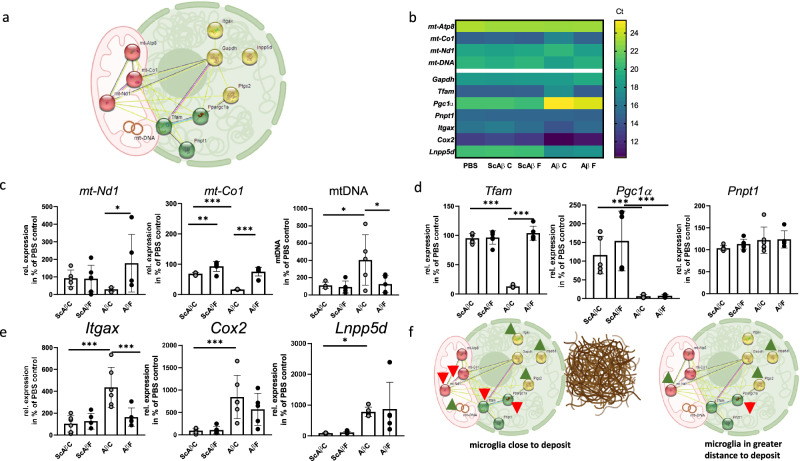
Gene expression patterning in nuclei and mitochondria from Aβ-deposit-exposed SIM-A9 cells in relation to deposit proximity. **a** To assess the effect of proximity of microglial cells to peptide deposits, eight genes were selected and their gene products analyzed in either nucleic or mitochondrial material sampled as described in Fig. 2. Three are nucleic encoded and affect mitochondrial homeostasis (green), two are mitochondrially encoded (red) and three genes (yellow) are not fundamentally related to mitochondrial function but have been described to respond to proximity to Aβ deposits. Gapdh and mt-Atp8 served as housekeeping controls. **b** Quantification of mitochondrial and nucleic mRNAs and mt-DNA. Mean of uncorrected threshold cycles are shown as a heat map. **c** Respective mt-RNAs were quantified from mitochondrial samples and related to mt-Atp8. The amount of mt-DNA was normalized to Gapdh mRNA values. **d**, **e** Nucleic mRNAs were quantified from nucleic samples and related to Gapdh in two independent experiments (*n* = 3 each; single values were excluded if outlier analysis indicated an outlier (ROUT, Q = 1%)). Values are expressed as mean + SD. Statistical analysis was performed by one-way ANOVA followed by Sidak’s multiple comparison post-test (***p < 0.001; **p < 0.01; *p < 0.05). **f** Summary of findings regarding mitochondrially and nucleic encoded genes in relation to proximity of amyloid deposits.

Nuclear and mitochondrial material was subjected to organelle-specific RT-qPCR (Fig. 3a), taking into account genes whose expression has previously been shown in the literature to be affected by amyloid plaque exposure. *Gapdh* and *mt-Atp8* showed no changes upon exposure to either scrambled peptide deposits or Aβ aggregates, irrespective of the distance to the deposited material.

Most of the investigated gene products were only differentially regulated in cells in close proximity to Aβ aggregates (Fig. 3b; C = close) and were not affected by the scrambled peptide administration. For example, mitochondrially encoded NADH:ubiquinone oxidoreductase core subunit 1 (mt-Nd1) and cytochrome c oxidase subunit 1 (mt-Co1) were both reduced in mitochondria derived from cells close to active peptide deposits (Fig. 3c, *p* = 0.0260 and < 0.0001). Additionally, mt-DNA amount was increased in samples derived from mitochondria in close proximity to Aβ peptide deposits (*p* = 0.0173). The peroxisome proliferator-activated receptor gamma coactivator 1α (Pgc1α) mRNA was strongly reduced, regardless of whether the nucleic material was obtained far (F) or close (C) from the Aβ-deposits (Fig. 3d, ScAβC versus AβC *p* = 0.0012 and ScAβF versus AβF *p* < 0.0001).

Next, we assessed expression of nucleic encoded genes that are not fundamentally involved in mitochondrial maintenance and homeostasis but have been found to be responsive to Aβ and are pivotal for microglial function: Itgax (integrin subunit alpha X; codes for CD11c), inositol polyphosphate-5-phosphatase (Inpp5d), and cyclooxygenase 2 (Cox2, Ptgs2). Itgax mRNA increased selectively in close proximity to deposits (Fig. 3e, *p* < 0.0001), while the other two gene products were increased independently from distance to deposit.

## Discussion

### Technical applicability of the subcellular sampling system

In the area of single cell-based technology, it is mandatory to be able to also collect intact material from single cells and probably even more – subcellular regions. We here explored the Single Cellome™ System SS2000 to investigate local effects of Aβ deposits (hallmarks of AD) on microglial mitochondria. While the system offers carbon dioxide supply to be integrated, we chose the option to work with CO_2_-independent culture medium. This ascertained a stable viability of the cells during samplings conducted within one hour (allowing about nine to twelve samplings). The system offers a range of fluorescent labels to be used for identification of subcellular regions. In the here presented experiments, DAPI and Lumitracker Mito Red were applied. By titrating the concentration of the mitochondrial stain, a threshold was experienced that—while cells were still clearly visibly stained – did not allow the system to identify the fluorescence for sampling. However, this was in a range of 12.5–2.5% of the stain concentration suggested by the manufacturers of comparable mitochondrial stains (minimal usage of 12.5 nM instead of 500–100 nM). Using only 25 nM of the Lumitracker Mito Red stain was sufficient to identify and extract mitochondrial material. This is of importance because of the potential photosensitizing effects such stains have been reported to show when administered at higher or comparable concentrations^17,18^.

Mitochondria can make up approximately 35% of the cell volume^19^; however, due to physiological conditions or pharmacological treatment, this volume can even increase up to 40-50%^20^. When calculating the amount of mitochondrial material aspirated from SIM-A9 cells, approximately 25% of the cellular content was sampled, taking into account the measured cell diameter and assuming a perfect spherical shape of the cell. The finding that red fluorescence was exclusively found in mitochondrial samples and not in nucleic material, underlines purity of the sample. However, a certain signal derived from excitation for DAPI staining was observed in mitochondrial material. This might hint at cross-contamination with nucleic material but it might also derive from staining of mitochondrial nucleoids as has been described before^16,21^. This is supported by the fact that only minor signals could be observed for the nucleus-encoded gene *Gapdh* in mitochondrial lysates while the signal for mt-Atp8 was rather strong. This finding for *Gapdh* could be also confirmed on the protein level by Western blotting (Suppl. Figure 3b and 4). However, contamination of the collected nuclear or mitochondrial material with other cellular material cannot be fully excluded. We tried to detect Calnexin as an endoplasmic reticulum (ER) marker in both fractions and did observe a respective band of about 90 kDa (Suppl. Figure 3b). This indicates that co-collection of ER material cannot be avoided, probably due to the wide and branched distribution of this organelle throughout the cell. Nevertheless, oxygen consumption could also only be detected in mitochondrial samples and be blocked to a high extent by antimycin A. Oxygen consumption in addition points at preserved functionality of the aspirated mitochondria; however, it is difficult to estimate the exact grade of functionality. More traditional methods for isolation of mitochondria via centrifugation take much longer than the direct sampling (e.g., 90 min^22^) and thus would not allow a side-by-side comparison of both methods with material derived from the same cell samples. Nevertheless, in sum our data revealed that sampling of mitochondria was feasible and delivered material with both, purity (in regard to nucleic contamination) and functionality, and comparable content of biological material. A limitation of our study is that we did not assess cellular viability after the extraction procedure. However, as the capillary diameter is much larger with 3 µm as compared to nanoscale extraction tools (openings in the range of 50–400 nm^23^,), we assume that the cells were damaged due to severe membrane disruption. This would not allow longitudinal sampling from one cell as has been demonstrated for nanoscale tools, which only lead to minimal disruptions (e.g^24^: puncturing of nucleus: >60% living cells). Nevertheless, using the microscale extraction method that we present here might also bear advantages: (1) mitochondria (for example, rodent smooth muscle cell mitochondria: 0.90 ± 0.20 µm in length and 0.63 ± 0.12 µm in width^25^) might be affected by flow stress due to too small pipette diameter. (2) Sampling of multiple organelles might better allow conducting functional assays such as the oxygen consumption assay, while for high sensitivity assays with amplification steps, such as RT-qPCR, material in the pL range from nanoscale isolation is sufficient. (3) The extraction chamber as well as the sample collection chamber of the commercial instrument are temperate (37 and 4°C). This allows keeping the cells in an optimal state during the process and immediately protects the samples after extraction. This might not always be the case in custom-built devices. For example, Chen et al. ^15^. described that while cells were kept at 37°C before and after the extraction process, the extraction via Live-seq itself was performed at room temperature.

### Spatial resolution of Aβ-deposit evoked damage in microglia

After confirming that characteristic subcellular material could be collected, SIM-A9 cells were exposed to Aβ-based plaque-like deposits^26^. The scrambled peptides which are biologically inactive, surprisingly also tended to built deposits after seven days of incubation. It was reported that while a prediction algorithm did not allocate amyloidogenic regions to either scrambled or reverse variants of Aβ, the peptides nevertheless formed fibrils^27^. However, ThT fluorescent signal was lower at 48 h of incubation for both as compared to wild type Aβ and – even more importantly – toxic effects on rat primary rat neurons were only minimal when compared to the wild type peptide after seven days of incubation. This is confirmed by the here presented ThT assay data and toxicity measures. One limitation of our study might be that we used aggregates of pure Aβ peptides. Plaques occurring in the brain of patients, however, comprise a wide variety of other proteins that also lead to distinguishable plaque subtypes. Complement and inflammatory response proteins as well as proteins from the lipid transport and metabolism section, proteins involved in blood coagulation and hemostasis and many others can be found – depending on the methods used for analysis (reviewed in ref. ^28^). For example, attached fibrinogen seems to be directly involved in microglia activation via fibrinogen-Cd11b binding as shown in 5xFAD AD model mice^29^. Thus, the here used peptide deposits only provide a simplified model of plaques to demonstrate feasibility of combined subcellular sampling and spatial resolution in response to Aβ-deposits. In close vicinity to the deposits, however, we found that oxygen consumption was reduced in mitochondria. In 5xFAD mice, for example, already at the age of two months progressive alterations of mitochondrial morphology and function such as oxygen consumption was observed in hippocampus^30^. This is coincident with timing of plaque deposition in the rather aggressive disease model and underlines the early impact Aβ deposits have on energy homeostasis in the brain. Microglia per se have a high energy-demand to maintain immune functions. In the resting state, they rely on OXPHOS to gain ATP; however, they can switch to anaerobic glucose usage in case of being stressed or activated^31,32^.This would coincide with reduced oxygen consumption as seen in the SIM-A9 cell-derived mitochondria in contact to Aβ deposits.

### Locally affected gene expression pattern

Expression of mitochondrially encoded genes was affected only in cells in close proximity to the Aβ deposits. Such downregulation of *mt-Nd1* has been previously described in BV-2 cells exposed to Aβ oligomers^33^ with an effect size of 40%. We observed a much stronger reduction (70%), which could be explained at least in part by local extraction. The *mt-Co1*, which was reduced by more than 80%, was also shown to be reduced in cells after Aβ administration, as were nucleic encoded mitochondrial transcription factor A (*Tfam*) mRNA levels^34^. The *Pgc1α*-mRNA was strongly reduced, regardless of whether the nucleic material was obtained far or close from the Aβ-deposits. This is consistent with the finding that a reduction in the expression of this gene is an event that occurs, for example, at an early age in AD mouse models^35^, indicating a rapid response that might not need the highly concentrated localized deposits and preceding mitochondrial damage. Finally, mt-DNA amount was increased in samples derived from mitochondria in close proximity to Aβ peptide deposits. Most reports indicate a reduction of mt-DNA in material derived from patients with AD or other neurodegenerative disorders^36^. However, defects in the fission machinery might also lead to a disturbed distribution of newly replicated mt-DNA nucleoids and subsequently to accumulation^37^. Moreover, the mt-DNA amount of a cell is determined by its energy demand and thus, the observed increase might be interpreted as an early attempt of the mitochondrial machinery to compensate for decreased energy provision resulting from altered gene expression.

Concerning nucleic encoded genes that are not fundamentally involved in mitochondrial maintenance and homeostasis but have been found to be responsive to Aβ, CD11c has been identified as a key marker for disease-associated microglia (DAM) and to be expressed in proximity to plaque foci^38^. Inpp5d was found to correlate with plaque density in human brain and expressed in plaque-associated microglia in a previous study^39^; however, distance to plaque was not taken into account. Cox-2 deficiency has been found recently to influence microglial density and morphology in mice^40^ and treatment of BV-2 microglia with Aβ led to increase of Cox-2 expression via NFκB signaling^41^. Thus, a global upregulation of the latter two genes coincides with these reports.

## Conclusion

Our research demonstrates the feasibility of extracting intact, functional mitochondrial material using the Single Cellome™ System SS2000. This commercial system allowed to first visualize mitochondria in single cells using confocal microscopy and second to decipher mitochondrial properties and function. This is a huge improvement regarding other methods, where mitochondria need to be sampled from thousands to millions of cells or from bulk tissue.

By applying the system to an investigation of Aβ-deposit-derived changes in nucleic and mitochondrial gene expression in microglia, a clear distinction due to the relative positioning of the cells was found (Fig. 3f). In future, this system might be used for sampling other particle-shaped organelles and it might be considered that mitochondria that can be collected in such rapid, function-sparing, and defined manner might also serve as a valuable source for mitochondrial transfer experiments^42^. Keeping in mind that a recent exciting paper from the Picard group^43^ shows distinct mitochondrial profiles in different brain areas, this method might allow digging even deeper in elucidating cell-specific mitochondrial function between neuronal populations, glia cells, and astrocytes. In addition, the system also could allow sampling selectively from cells containing pre-dominantly fragmented versus from such with fused mitochondria and exploring their differential gene expression.

## Methods

### SIM-A9 Cell Culture

Spontaneously immortalized mouse microglia (SIM-A9, BIOZOL Diagnostica Vertrieb GmbH, KER-END001)^44^ were cultivated in 10 cm culture dishes (Sarstedt AG & Co. KG) in a CO_2_ incubator (Forma™ STERICULT CO_2_ Incubator, Thermo Fisher Scientific Inc.) at 37 °C, 95% humidity, and 5% CO_2_. The culture medium consisted of phenol red-free Dulbecco’s Modified Eagle Medium/Nutrient Mixture F-12 (DMEM:F-12), supplemented with 10% v/v heat-inactivated fetal bovine serum (Gibco®, Life Technologies), 5% v/v heat-inactivated donor horse serum (Gibco®, Life Technologies), 1% v/v Penicillin–Streptomycin (Sigma-Aldrich), and 1% v/v L-Glutamine (Sigma-Aldrich). Upon reaching 80% confluence, the cells were passaged. Therefore, the conditioned medium was aspirated, and the cells were washed with 5 ml of pre-heated phosphate-buffered saline (PBS, 37 °C). Subsequently, 2.5 ml of Trypsin/EDTA-solution (Sigma-Aldrich) were added for 5 min at 37 °C, 95% humidity, and 5% CO_2_. The trypsin reaction was halted by adding 7.5 ml of culture medium. Following resuspension, the cells were transferred to a 50 ml CELLSTAR®, BLUE SCREW CAP Tube (Greiner Bio-One International GmbH) and centrifuged in a Megafuge 1.0 R (Heraeus) for 6 min at 600xg. The cell supernatant was removed, and the cell pellet was suspended in 20 ml of culture medium. Finally, 10 ml of suspended cells were distributed evenly between two culture dishes. Work with the SIM-A9 cell culture was conducted within a sterile biosafety cabinet (MSC-Advantage™, Thermo Fisher Scientific Inc.). For experiments, aliquots from cell suspension following the termination of the trypsin reaction were diluted with PBS and cells counted using the Cell Scepter TM 3.0 (Merck KGaA). Twenty-thousand cells per well were seeded in black, glass bottom 96-well plates (Greiner Bio-One GmbH).

### Staining of Nuclei with DAPI

The 4’,6-diamidino-2-phenylindole (DAPI; Sigma-Aldrich, D9542) stock solution (1 mg/mL) was diluted 1:2000 with 1×PBS and 50 μL were added per well. The plate was incubated in darkness for 10 min at 37 °C using the Thermomixer comfort (Eppendorf AG). Following incubation, the DAPI solution was aspirated, and the cells underwent two washing steps with 200 μL of 1×PBS each. Subsequently, staining with LumiTracker Mito Red CMXRos was carried out.

### Staining of Mitochondria with LumiTracker Mito Red CMXRos

The LumiTracker Mito Red CMXRos (1 mM, Lumiprobe GmbH, 2251) stock solution was diluted 1:40,000 in pre-warmed CO_2_-independent culture medium (Gibco®, Life Technologies). 50 μL of the prepared solution were added per well, followed by a 15 min incubation at 37 °C in the Single Cellome™ System 2000 (Yokogawa Electric Corporation; Tokyo, Japan). After incubation, organelle material extraction was performed.

### Peptide Aggregation

For peptide aggregation, aliquots containing 40 µL of 350 µM of Aβ1-42 or scrambled Aβ1-42 (both Bachem, 4014447 and 4104168) in sterile 1xPBS were incubated at 37°C as described previously^26^. To induce aggregation, these aliquots were subjected to 50 pipette strokes using 20 µL filter-pipette tips. The pipetting regimen commenced immediately on day 1, followed by repetitions after 48 hours on day 3, and on day 6. After 168 hours (on day 7 from the start of incubation), the resulting deposits of Aβ1-42 or scrambled Aβ1-42 were utilized for cell culture experiments. Aggregate formation within the Aβ1-42 solution was confirmed by ThT assay (Supplementary Fig. 2a) and by assessing toxicity using Cell Titer Glo assay (Promega, G7570) (Suppl. Figure 2b). Toxicity was probably not elicited by the larger fibrillary aggregates that could be visualized during extraction but by oligomeric forms^45^. Ten µL of aggregated peptide solution or of 1xPBS were added to the cells (in 90 µL culture medium volume). The added peptide preparation or solvent were carefully mixed with the cultivation medium by ten pipette strokes with 50 µL volume. The cells where subsequently cultivated for 24 h in an incubator (Forma™ STERICULT CO_2_ Incubator, Thermo Fisher Scientific Inc.) at 37 °C, 95% humidity, and 5% CO_2_.

### ThT assay

ThT-solution was freshly diluted to 10 µM with PBS (Thioflavin T, Sigma Aldrich, T3516). Thirty µl of peptide-containing solution were mixed with 55 µl of ThT solution in black 96 well plates and fluorescence measured at 37°C with Ex/Em=440 nm/484 nm (Fluostar Omega, BMG Labtech).

### Measuring Cell-Peptide-Deposit Distance

Using the built-in custom ruler tool integrated with the pixel-to-metric unit translator of the Single Cellome™ System 2000, the distance between two deposits was measured by delineating a line from the edge of one deposit to the edge of the other. The midpoint of this line was identified as the farthest point, while the starting points of the line were defined as the nearest point. Subsequently, cells were categorized into three groups: those far from the deposits, situated more than 10 µm away from the midpoint between two deposits; those near the plaques, within 10 µm of the edge of the deposit; and those in between. Only cells categorized as far or near to a deposit were selected for organelle harvesting.

### Organelle Material Extraction Using Single Cellome™ System 2000

Mitochondrial and nuclear materials were extracted employing the Single Cellome™ System 2000. The Single Cellome™ System 2000 was programmed to manual entry of the cell and automatic discharge of collected material into a collection plate. The collection loader was cooled to 4 °C, while the cell incubation loader was heated to 37 °C. The microscope channels were configured for fluorescence visualization, with 561 nm Exc/ 617 nm Em (red fl.) or 405 nm Exc/ 447 nm Em (blue fl.). The objective lens was adjusted to a magnification of “40x Dry.” Upon selection of the respective cell, the tip was inserted into the cell with controlled velocity (10 μm/s) and pressure (1 Pa). The material was extracted by changing the pressure to −10 Pa for 5 s and automatically discharged into the collection plate (MicroAmp™ optical 96-well reaction plate, Applied Biosystems™), pre-filled with 6 μL of sterile 1×PBS for use in RT-qPCR and qPCR or 6 μL of sterile measurement buffer (250 mM sucrose (Sigma-Aldrich), 15 mM KCl (Carl Roth GmbH + Co. KG), 5 mM MgCl_2_ (Carl Roth GmbH + Co. KG), 30 mM K_2_HPO_4_ (Carl Roth GmbH + Co. KG), 50 mM succinate (Sigma-Aldrich), pH 7.4) for the Oxygen Consumption Assay. For each sample, material from three cells from the same well was extracted and pooled. The total extraction process took approximately 4 minutes, with several distinct steps. The identification of a suitable cell required around 30 seconds, followed by 2 to 3 min for internal machine positioning and loading of the extraction tips. The subsequent alignment of the tip relative to the cell took an additional 20–30 s. Finally, the actual extraction of the cellular material was completed in approximately 10 s.

To ascertain that the extracted material did not vary in general amount of to be measured molecules (DNA, RNA, and protein), we assessed the respective data by using the absorption at 260 nm for nucleic acids (Nanodrop) and the ProteOrange kit (Lumiprobe GmbH, 14102) for measuring small amounts of protein. All sample means did not differ statistical from each other or from the control (Supplementary Fig. 3a).

### Fluorescence Analysis of Extracted Organelle Samples

The extracted material was transferred to a black 384-well plate with transparent bottom (Greiner Bio-One GmbH), and supplemented with 4 μL of nuclease-free water. Fluorescence signals were measured using the FLUOstar® Optima microplate reader (BMG Labtech GmbH), with 485 nm Exc/ 520 nm Em (DAPI blue fl.) or with 540 nm Exc/ 580 nm Em (LumiTracker Mito Red CMXRos, red fl.).

### Reverse Transcription quantitative Polymerase Chain Reaction (RT-qPCR) and qPCR

Six μL of samples were combined with the Cell lysis mix (Luna ® Cell Ready One-Step RT-qPCR Kit, New England Biolabs, E3030S), excluding DNAseI, resulting in a total volume of 40 μL. 32 μL were transferred into a new vial and the lysis protocol proceeded according to the manufacturer’s instructions. For qPCR reactions, the remaining 8 μL of sample lysis mix underwent the manufacturer’s protocol, substituting DNAseI with 10 μM RNase A (Carl Roth GmbH + Co. KG) and incubation for 10 min at 37 °C. RT-qPCR was carried out with Luna ® Cell Ready One-Step RT-qPCR Kit (New England Biolabs) and qPCR with primaQUANT SYBRGreen qPCR Blue with ROX (Steinbrenner Laborsysteme GmbH) adhering to the manufacturer’s guidelines. The sample volume was adjusted to 20 μL per sample and a primer concentration of 0.4 μM (Qiagen or produced by EurofinsMWG). The reactions were carried out on a StepOnePlus™Real-Time PCR System (Applied Biosystems™). For primer sequences see Table 1.

**Table1:** Primer sequences

Primer	Sequence 5ʹ- > 3ʹ	Reference
mt-Atp8_for	GGCACCTTCACCAAAATCAC	^46^
mt-Atp8_rev	TTGTTGGGGTAATGAATGAGG	
mt-Co1_for	CCTAGATGACACATGAGCAAAAG	(forward primer modified)^47^
mt-Co1_rev	AGCGTCGTGGTATTCCTGAAA	
mt-Nd1_for	ACGCTTCCGTTACGATCAAC	^46^
mt-Nd1_rev	ACTCCCGCTGTAAAAATTGG	
mt-DNA_for	CTAGAAACCCCGAAACCAAA	^48^
mt-DNA_rev	CCAGCTATCACCAAGCTCGT	
mGapdh	QuantiTect Primer Assay (Qiagen)	
Tfam_for	CCAAAAAGACCTCGTTCAGC	^49^
Tfam_rev	ATGTCTCCGGATCGTTTCAC	
Pnpt1_for	AATCGGGCACTCAGCTATTTG	PrimerBank ID 12835817a1^50^
Pnpt1_rev	CAGGTCTACAGTCACCGCTC	
PGC1α_for	CGGAAATCATATCCAACCAG	^51^
PGC1α_rev	TGAGGACCGCTAGCAAGTTTG	
Cox2_for	GGGTGTGAAGGGAAATAAGG	^52^
Cox2_rev	TGTGATTTAAGTCCACTCCATG	
Itgax_for	CCATGCTGGCTGTAGATGACC	^53^
Itgax_rev	GTCATCCTGGCAGATGTGGTC	
Inpp5d_for	GAGCTACTTTCCAGAGCCG	^54^
Inpp5d_rev	CACAATTCCGGAACAGCACG	

### Oxygen Consumption Assay

The Oxygen Consumption Rate Assay Kit (Cayman Chemicals, CAY 600800-96) was performed in accordance with the manufacturer’s guidelines with the reagent volumes adjusted to 40 μL in a black 384-well plate with transparent bottom (Greiner Bio-One GmbH), using 6 μL of organelle material in measurement buffer. Antimycin A (1 μM; Cayman Chemicals) was employed for inhibition, while glucose oxidase was utilized as a positive control for oxygen consumption. Fluorescence signal was measured by the FLUOstar® Optima microplate reader (BMG LABTECH) with an excitation wavelength of 380 ± 12 nm, and an emission wavelength of 630 ± 12 nm. The linear phase from the kinetic measurement was used for calculation of oxygen consumption.

### Western blotting

Proteins were subjected to 10% SDS PAA gels. As control, 40 µg (lysate high, Lh) and 14 µg (lysate low, Ll) of protein derived from lysed SIM-A9 cells was used and 14 µg of protein from extracted material (nuclear or mitochondrial). Proteins were transferred to nitrocellulose membrane and the membrane was blocked with I-block solution (0.2% in PBS) (Thermo Fisher Scientific) including 0.05% Tween 20 (AppliChem). Primary antibody incubation took place overnight at 4 °C with anti-Calnexin (Cnx; Cloud-Clone Corp. - Cat. PAA280Mu01, dilution 1:1000) or anti-Gapdh (AB_561053, Cell Signaling Technology, dilution 1:1000) in combination with HRP-labeled secondary antibodies (1:3000, Thermo Fisher Scientific). Signals were detected after incubation with SuperSignal West Femto chemiluminescent substrate (Thermo Fisher Scientific, 34095) using a CCD-camera imaging system (Stella Camera, Raytest, Straubenhardt).

### Statistics and Reproducibility

Data were obtained from at least two independent experiments as indicated in the figure legends. All statistical analyses were conducted using GraphPad Prism 6 or 8 software (GraphPad Software). All data are presented as mean ± standard deviation. Statistical significance of differences between two groups was determined using two-tailed Student’s t-tests. One-way analysis of variance (ANOVA) was used for three or more groups and a post-hoc pairwise comparison as indicated. Outlier analysis was performed with GraphPadPrism (ROUT 1%). A *p*-value < 0.05 was considered statistically significant.

### Images

Schematics were created by using Biorender.

### Reporting summary

Further information on research design is available in the Nature Portfolio Reporting Summary linked to this article.

## Supplementary information

Supplementary Information

Description of Additional Supplementary File

Supplementary data 1

Reporting Summary

## Data Availability

All data are available in the main text, Methods or Supplementary Data (source data for figures and analysis are provided in Suppl. Data 1).
