# Supplementary material for: A subcellular sampling instrument allows spatial resolution of amyloid deposit-derived organelle-specific effects in microglia
Source: Commun Biol. 2025 Jan 3;8:3. doi: 10.1038/s42003-024-07405-w (PMC11699115; doi:10.1038/s42003-024-07405-w)

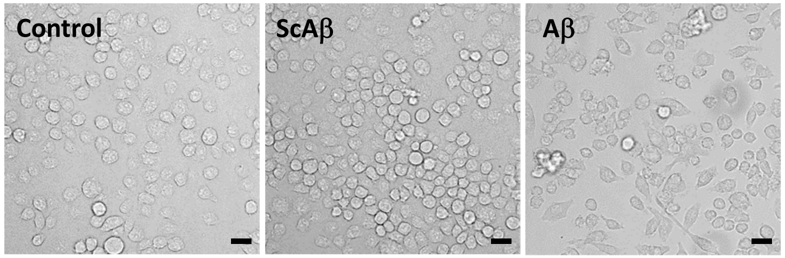

Suppl. Fig. 1. Cell imaging after peptide exposure. Cells were exposed to pre-aggregated peptides or solvent control (PBS). Sample images were taken after 24h. Scale bar: 25 µm. Sc: scrambled.

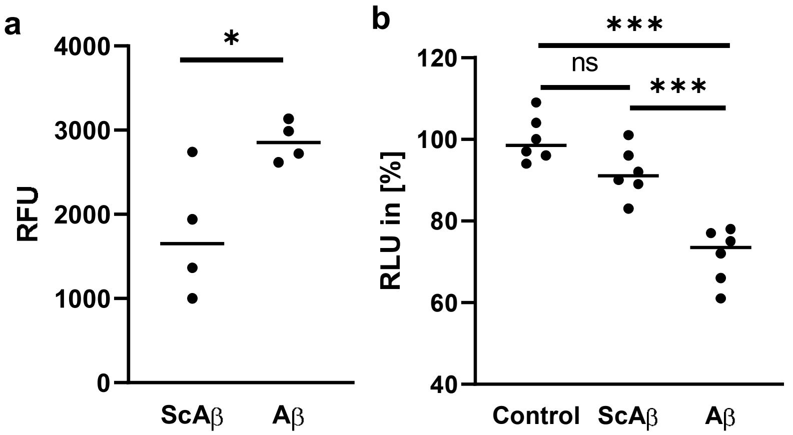

Suppl. Fig. 2. Characterization of Aβ peptide material after incubation.
a, After 168 hours (on day 7 from the start of incubation), aliquots of the peptide solutions were subjected to ThT assay. The relative fluorescence (RFU) is proportional to the formed aggregates. Respectively incubated PBS served as a background control and was subtracted from the values for peptide solutions. Significance was tested by Student's two-sided unpaired t-test (*, $p < 0.05$). b, Cells were incubated as described with aggregates from ScAβ or Aβ and ATP content in the cell lysate as an indicator of metabolic activity/ viability assessed via Cell Titer Glo Assay (Promega). Relative luminescence units are presented as % of control (PBS). Two independent experiments were performed. Values are expressed as mean. Statistical analysis was performed by one-way ANOVA followed by Sidak's multiple comparison post-test (***, $p < 0.001$; ns > 0.05).Sc: scrambled.

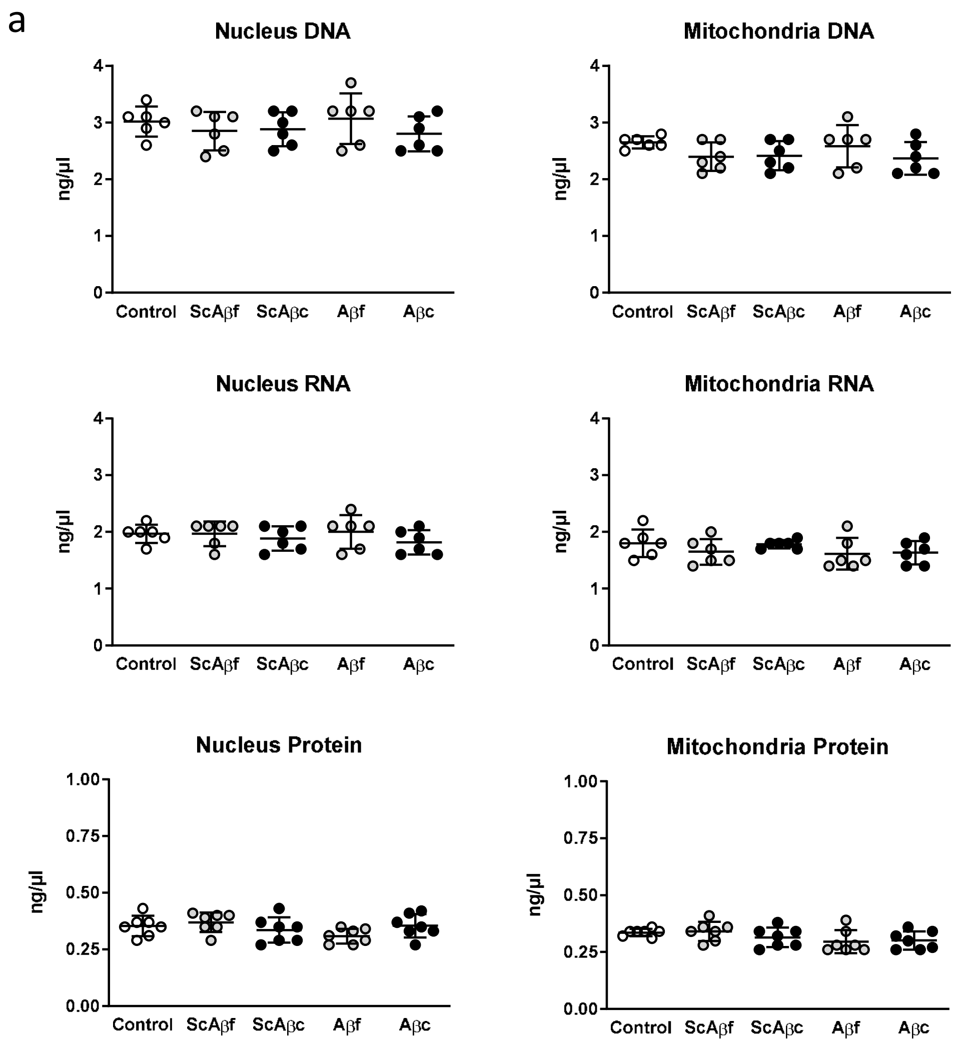

Suppl. Fig. 3. Comparison of extracted cellular material. a) After staining with Lumitracker Mito Red and DAPI, cells were used for sampling of mitochondrial or nucleic material using the Single Cellome™ System SS2000. RNA and DNA of either nucleic or mitochondrial material from three cells pooled was measured by absorption at 260nm (Nanodrop spectrophotometer). Protein content was assessed by using the ProteOrange Kit following the manufacturer's instructions. Mean values ± SD are presented (n=6 per group). One-way ANOVA with Sidak's multiple comparison post-test revealed no statistical differences. **b)** Samples of extracted material were pooled (14 µg protein each) via chloroform-methanol precipitation and subjected to Western blot analysis. Lysate proteins of 40 µg (lysate high, Lh) and 14 µg (lysate low, Ll) served as positive controls. Three exemplary samples are shown each for nuclear material (N) or mitochondrial material (M). Primary antibodies were directed against Calnexin (Cnx; Cloud-Clone Corp. - Cat. PAA280Mu01, dilution 1:1000) or Gapdh (AB_561053, CellSignaling Technology, dilution 1:1000). P: PBS-treated; Sc: scrambled; c: close; f: far. The blot was cut for presentation, indicated as dashed lines (for the uncut blot, see Suppl. Fig.4).

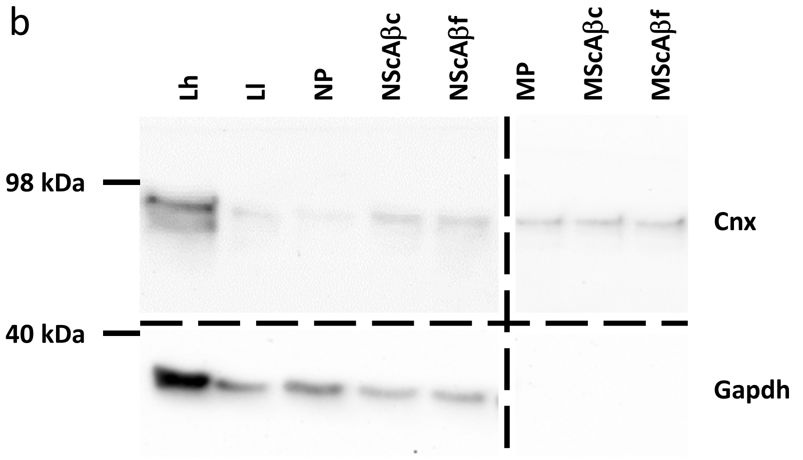

Suppl. Fig. 4. Uncropped Western blot for Suppl. Figure 3 and marker images. a) and **b)** show the upper part of the Western blot, which was used for detection of Calnexin (see Suppl. Figure 3b). **c)** and **d)** show the lower part, which was used for Gapdh detection. As a protein ladder pre-stained SeeBlue™ Plus2 (ThermoFisher) was used.

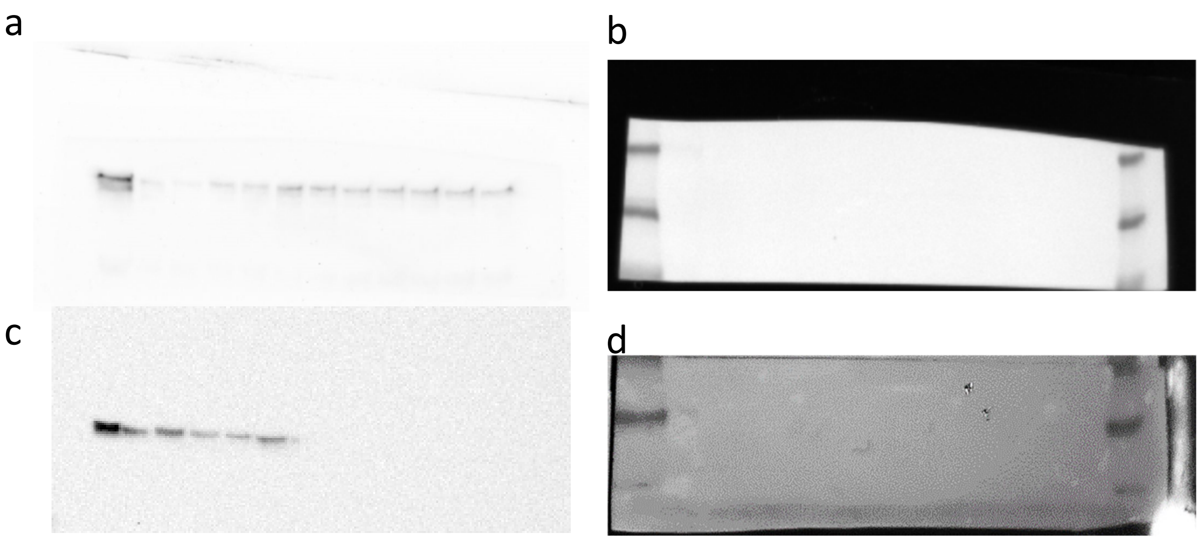

Supplement: Supplementary file 1 — Supplementary Information [file 42003_2024_7405_MOESM1_ESM.pdf]
