# Supplementary material for: A subcellular sampling instrument allows spatial resolution of amyloid deposit-derived organelle-specific effects in microglia
Source: Commun Biol. 2025 Jan 3;8:3. doi: 10.1038/s42003-024-07405-w (PMC11699115; doi:10.1038/s42003-024-07405-w)
Supplement: Supplementary file 4 — Reporting Summary [file 42003_2024_7405_MOESM4_ESM.pdf]

nature portfolio

Corresponding author(s): K. Endres

Last updated by author(s): Dec 2, 2024

Reporting Summary

Nature Portfolio wishes to improve the reproducibility of the work that we publish. This form provides structure for consistency and transparency in reporting. For further information on Nature Portfolio policies, see our Editorial Policies and the Editorial Policy Checklist.

Statistics

For all statistical analyses, confirm that the following items are present in the figure legend, table legend, main text, or Methods section.

n/a	Confirmed	
☐	☒	The exact sample size (n) for each experimental group/condition, given as a discrete number and unit of measurement
☐	☒	A statement on whether measurements were taken from distinct samples or whether the same sample was measured repeatedly
☐	☒	The statistical test(s) used AND whether they are one- or two-sided<br>*Only common tests should be described solely by name; describe more complex techniques in the Methods section.*
☐	☒	A description of all covariates tested
☐	☒	A description of any assumptions or corrections, such as tests of normality and adjustment for multiple comparisons
☐	☒	A full description of the statistical parameters including central tendency (e.g. means) or other basic estimates (e.g. regression coefficient) AND variation (e.g. standard deviation) or associated estimates of uncertainty (e.g. confidence intervals)
☒	☐	For null hypothesis testing, the test statistic (e.g. F, t, r) with confidence intervals, effect sizes, degrees of freedom and P value noted<br>*Give P values as exact values whenever suitable.*
☒	☐	For Bayesian analysis, information on the choice of priors and Markov chain Monte Carlo settings
☒	☐	For hierarchical and complex designs, identification of the appropriate level for tests and full reporting of outcomes
☒	☐	Estimates of effect sizes (e.g. Cohen's d, Pearson's r), indicating how they were calculated

Our web collection on statistics for biologists contains articles on many of the points above.

Software and code

Policy information about availability of computer code

Data collection	Single Cellome™ System 2000 (Yokogawa), software Fluostar Optima, software StepOne plus cycler
Data analysis	Excel 2016, GraphPadPrism 6 and 8

For manuscripts utilizing custom algorithms or software that are central to the research but not yet described in published literature, software must be made available to editors and reviewers. We strongly encourage code deposition in a community repository (e.g. GitHub). See the Nature Portfolio guidelines for submitting code & software for further information.

Data

Policy information about availability of data

All manuscripts must include a data availability statement. This statement should provide the following information, where applicable:

- Accession codes, unique identifiers, or web links for publicly available datasets
- A description of any restrictions on data availability
- For clinical datasets or third party data, please ensure that the statement adheres to our policy

Data availability statement has been inserted.

Research involving human participants, their data, or biological material

Policy information about studies with human participants or human data. See also policy information about sex, gender (identity/presentation), and sexual orientation and race, ethnicity and racism.

Reporting on sex and gender	The cell line SIM-A9 is a spontaneously immortilized line derived from a culture pooled from mouse pups. No information on sex of the herefore used animals is provided by the vendor.
Reporting on race, ethnicity, or other socially relevant groupings	NA
Population characteristics	NA
Recruitment	NA
Ethics oversight	NA

Note that full information on the approval of the study protocol must also be provided in the manuscript.

Field-specific reporting

Please select the one below that is the best fit for your research. If you are not sure, read the appropriate sections before making your selection.

☒ Life sciences ☐ Behavioural & social sciences ☐ Ecological, evolutionary & environmental sciences

For a reference copy of the document with all sections, see nature.com/documents/nr-reporting-summary-flat.pdf

Life sciences study design

All studies must disclose on these points even when the disclosure is negative.

Sample size	No sample size calculation was performed in advance. Sample size was estimated on comparable studies performed before, e.g. on biochemical properties or mRNA quantitation (RT-qPCR).
Data exclusions	Data were excluded from calculations if outlier analysis indicated an outlier (GraphPad Prism; ROUT, Q = 1%). Samples were excluded when successful material aspiration was not ascertained.
Replication	Independent experiments were performed with differing passages of cells. None of the single experiments indicated non-reproducibility.
Randomization	Wells with cells were not randomized to treatment (Abeta or scrambled peptide). However, order of wells was changed in between experiments to ascertain that position effects were excluded.
Blinding	Blinding was not possible due to the delicate technique of the device. It was not possible to train more than one person in the short time frame of the access to the device in-depth appropriately. However, data calculation and statistics were performed under supervision to exclude potential bias.

Reporting for specific materials, systems and methods

We require information from authors about some types of materials, experimental systems and methods used in many studies. Here, indicate whether each material, system or method listed is relevant to your study. If you are not sure if a list item applies to your research, read the appropriate section before selecting a response.

Materials & experimental systems

n/a	Involved in the study	
☐	☒	Antibodies
☐	☒	Eukaryotic cell lines
☒	☐	Palaeontology and archaeology
☒	☐	Animals and other organisms
☒	☐	Clinical data
☒	☐	Dual use research of concern
☒	☐	Plants

Methods

n/a	Involved in the study	
☒	☐	ChIP-seq
☒	☐	Flow cytometry
☒	☐	MRI-based neuroimaging

Antibodies

Antibodies used	anti-Calnexin (Cnx; Cloud-Clone Corp. - Cat. PAA280Mu01, dilution 1:1000)/ anti-Gapdh (AB_561053, CellSignaling Technology, dilution 1:1000)
Validation	anti-Calnexin:<br>Distributor homepage (Biozol) Species Reactivity: Mouse<br>Application: ICC, IHC, IP, WB<br><br>anti-GAPDH<br>Species Reactivity:<br>Human, Mouse, Rat, Monkey, Bovine, Pig<br>Product Usage Information on the vendor's homepage<br>Western Blotting 1:1000

Eukaryotic cell lines

Policy information about cell lines and Sex and Gender in Research

Cell line source(s)	SIM-A9 cells BIOZOL Diagnostica Vertrieb GmbH
Authentication	The cell line was authenticated by reactivity to stimuli (e.g., TNFalpha secretion upon LPS administration) and by staining of respective glial markers (such as Iba1).
Mycoplasma contamination	Cells were not tested for mycoplasma. Regularly, DAPI staining is performed which would at least report on severe contamination.
Commonly misidentified lines<br>(See ICLAC register)	No commonly misidentified cell line was used.

Plants

Seed stocks	NA
Novel plant genotypes	NA
Authentication	NA
